# Patient and public involvement in palliative care research: What works, and why? A qualitative evaluation

**DOI:** 10.1177/0269216320956819

**Published:** 2020-09-11

**Authors:** Halle Johnson, Margaret Ogden, Lisa Jane Brighton, Simon Noah Etkind, Adejoke O Oluyase, Emeka Chukwusa, Peihan Yu, Susanne de Wolf-Linder, Pam Smith, Sylvia Bailey, Jonathan Koffman, Catherine J Evans

**Affiliations:** 1 Cicely Saunders Institute of Palliative Care, Policy & Rehabilitation, Florence Nightingale Faculty of Nursing, Midwifery & Palliative Care, King’s College London, London, UK; 2 Patient and Public Contributor, Cicely Saunders Institute, King’s College London, London, UK; 3 Institute of Nursing, School of Health Professions, Zurich University of Applied Sciences, Winterthur, Switzerland; 4 Sussex Community NHS Foundation Trust, Brighton General Hospital, Brighton, UK

**Keywords:** Palliative care, consumer involvement, patient involvement, evaluation research

## Abstract

**Background:**

Public involvement is increasingly considered a prerequisite for high-quality research. However, involvement in palliative care is impeded by limited evidence on the best approaches for populations affected by life-limiting illness.

**Aim:**

To evaluate a strategy for public involvement in palliative care and rehabilitation research, to identify successful approaches and areas for improvement.

**Design:**

Co-produced qualitative evaluation using focus groups and interviews. Thematic analysis undertaken by research team comprising public contributors and researchers.

**Setting/participants:**

Researchers and public members from a palliative care and rehabilitation research institute, UK.

**Results:**

Seven public members and 19 researchers participated. Building and maintaining relationships, taking a flexible approach and finding the ‘right’ people were important for successful public involvement. Relationship building created a safe environment for discussing sensitive topics, although public members felt greater consideration of emotional support was needed. Flexibility supported involvement alongside unpredictable circumstances of chronic and life-limiting illness, and was facilitated by responsive communication, and opportunities for in-person and virtual involvement at a project- and institution-level. However, more opportunities for two-way feedback throughout projects was suggested. Finding the ‘right’ people was crucial given the diverse population served by palliative care, and participants suggested more care needed to be taken to identify public members with experience relevant to specific projects.

**Conclusion:**

Within palliative care research, it is important for involvement to focus on building and maintaining relationships, working flexibly, and identifying those with relevant experience. Taking a strategic approach and developing adequate infrastructure and networks can facilitate public involvement within this field.

**What is already known about the topic?**

● Public involvement in palliative care is impeded by limited evidence on the best approaches to use in populations affected by life-limiting illness.

**What this paper adds?**

● We provide a qualitative evaluation of an institutional-level strategy supporting patient and public involvement in palliative care and rehabilitation research.

● Public involvement in palliative care research requires a focus on building and maintaining relationships with careful consideration of emotional support when broaching sensitive topics; the ability to work flexibly with people living in complex and unpredictable circumstances; and an emphasis on involving people across the diversity of our field who have experience relevant to the specific research project.

**Implications for practice, theory or policy**

● Taking a strategic approach to public involvement within a research institute based on the above principles can facilitate involvement for people with life-limiting illness in research on palliative care and rehabilitation.

● Ongoing evaluation of approaches to public involvement is crucial to understand what works well and to minimise unintended consequences.

● A collaborative approach to public involvement in palliative care and rehabilitation research across organisations may help progress the quality, diversity and extent of public involvement within this field.

## Introduction

Public involvement is the process by which research is conducted with or by patients, carers or members of the public, to ensure research remains relevant to the populations in which it is conducted.^1^ This can include involving the public to identify research priorities, plan study designs, collect, analyse and interpret data, disseminate and/or implement research findings.^1^ Public involvement is increasingly considered a prerequisite for high-quality research, with the potential to improve research relevance, quality and impact.^1,2^ It is required by most research funders in the UK,^3,4^ and is becoming more common internationally.^5,6^

Public involvement within palliative care research can help to develop patient-focused research questions,^7,8^ aid recruitment to studies,^8^ and support dissemination of findings to a wider community.^7–9^ Yet, there is a lack of evidence on the best approaches to public involvement in palliative care. Accessing the views of those who may have advanced illness and considerable disability can be challenging, and involvement can be time consuming and resource-intensive for both researchers and public contributors.^10^ A recent review identified eight broad themes which facilitated and hindered successful involvement within this field.^11^ These included definitions and roles, values and principles, organisations and culture, training and support, networking and groups, perspectives and diversity, relationships and communication, and emotions and impact.^11^ However, most involvement in palliative care research to date has been consultative, rather than co-productive, and many of the approaches which have been evaluated have focused only on cancer-specific populations.^7,10–12^

**Figure 1.**
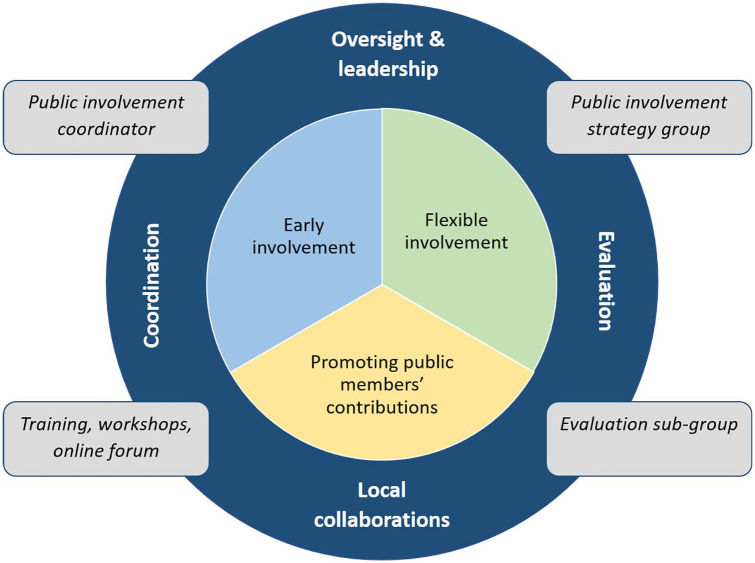
Summary of public involvement strategy (core principles and infrastructure).

Daveson et al. conducted a transparent expert consultation with palliative care patients, informal carers and researchers to better understand priorities for public involvement in palliative care.^13^ Stakeholders emphasised the following principles: early involvement; flexible involvement; and recognising and promoting the contribution of users.^13^ These principles formed the basis of a novel three-year (2017–2020) public involvement strategy at our palliative care and rehabilitation research institute. The strategy details a collaborative approach to how public involvement is implemented and evaluated to enhance research productivity, quality and clinical relevance. Alongside the core principles, the strategy commits to key infrastructure to support involvement, including a public involvement coordinator, public involvement training, and an online forum^14^ (see Figure 1).

Our aim was to evaluate this strategy for public involvement in palliative care and rehabilitation research, to identify successful approaches and areas for improvement. Importantly, this was not intended as an evaluation of ‘outcomes’ of public involvement as if it were a complex intervention.^15^ Rather, we sought to understand the positive and negative impacts of the strategy (its principles and infrastructure), from both the researcher and public perspective, to support continued development of public involvement in palliative care.

## Methods

### Design

#### Co-produced qualitative study

##### Co-production

We took a co-productive approach to this project, collaborating with three public contributors (MO, SB, PS) at all stages.^16^ By co-production, we mean that there was shared input and decision making in developing and undertaking the evaluation, as well as having shared power in the generation of knowledge which came from the evaluation.^16^ Specifically, public contributors were involved in the formation of project aims and objectives, protocol development, ethics application, development of topic guide, and were heavily involved in data analysis, interpretation and dissemination (see GRIPP2 checklist^17^ in Supplemental File 3).

##### Setting

A palliative care and rehabilitation research institute, UK, comprising approximately 50 researchers and 30 public members.

##### Participants

Institute public members were invited via email to participate in a focus group during a routine workshop in June 2018 (workshops typically comprise approximately 10 public involvement members), and researchers were invited via email to participate in a focus group during a routine meeting in July 2018 (typically comprising approximately 30 researchers). Convenience sampling was used to maximise parti-cipation.

##### Data collection

Focus groups for both researchers and public members were facilitated by members of the research team with experience of qualitative research (HJ, SE, AO, LB). Field notes were taken during, and following, the focus groups. Two interviews were also conducted with public members who were unable to attend the focus group due to illness and caring commitments. Focus groups and interviews were scheduled for one hour and followed a semi-structured topic guide, which was co-produced by researchers and public contributors.^16^ The topic guide explored participants’ experiences of involvement in relation to the strategy (Supplemental File 1). Written informed consent was obtained prior to audio-recording of the focus groups and interviews.

##### Analysis

Analysis was conducted jointly by researchers and public contributors within the research team.^16^ Focus group and interview data were transcribed verbatim and pseudonymised. We used NVivo 11 for data management.^18^ Data were analysed thematically,^19^ using the key principles of the public involvement strategy as an overarching framework, but coding inductively within these domains, and paying attention to novel themes not considered in the initial framework. To ensure rigour and trustworthiness, researchers (HJ, LB, SE, AO, PY, EC) independently examined transcripts to identify initial codes and then held a collaborative meeting with public contributors to refine the coding framework (Supplemental File 2). This framework was applied to the data by researcher (HJ), and double coding of a sub-section of the transcripts was completed by public contributor (MO), to check rigour of application and to ensure interpretation was not dominated by a researcher perspective. Where coding differed between researcher (HJ) and public contributor (MO), discrepancies were resolved through discussion. From coded transcripts HJ and MO identified overarching themes, incorporating public and researcher perspectives. At this stage we considered areas which converged, complemented, and were discrepant as well as instances of silence – where a theme or finding was identified in one group but not the other. Additionally, HJ and MO considered where involvement had worked well and where there were opportunities to enhance involvement. During interpretation we considered how the findings related to the recently developed National Standards for involvement^20^ as well as relevant theoretical models of involvement.^21^

##### Ethical considerations

Ethical approval was obtained from the King’s College London’s Research Ethics Committee (LRS-17/18-6473). Due to the research team’s relationship with potential participants, and to minimise any perceived pressure to participate, information sheets clearly communicated that participation was entirely voluntary. Although identifiable information on individual participants have been removed, participants were made aware that the institute would be identified when reporting the findings.

## Results

We conducted three focus groups with researchers (*n* = 19) and one focus group (*n* = 5), one face-to-face interview (*n* = 1), and one telephone interview (*n* = 1) with public members. Participant characteristics are provided in Table 1.

**Table 1.** Characteristics of researcher and public member participants, *N*.

	Researchers (*n* = 19)	Public members (*n* = 7)
Gender		
Female	15	5
Male	4	2
Ethnicity		
Asian or Black	5	1
Mixed	3	0
White British or White Other	11	6
Researcher Type		
Pre-doctoral researcher	9	-
Post-doctoral researcher	6	-
Lecturer/Senior Lecturer/Reader/Professor	4	-
Age (years)		
Up to 39	11	0
40–59	6	6
60+	0	1
Prefer not to say	2	0
Length of time in research		
Less than 1 year	2	-
1 year to 3 years	7	-
3 years to 5 years	2	-
Over 5 years	8	-
Length of time involved in the public involvement group		
Less than 1 year	-	1
1 year to 3 years	-	1
Over 3 years	-	5
Length of time involved in public involvement in research		
Less than 3 years	11	0
Over 3 years	8	7

**Figure 2.**
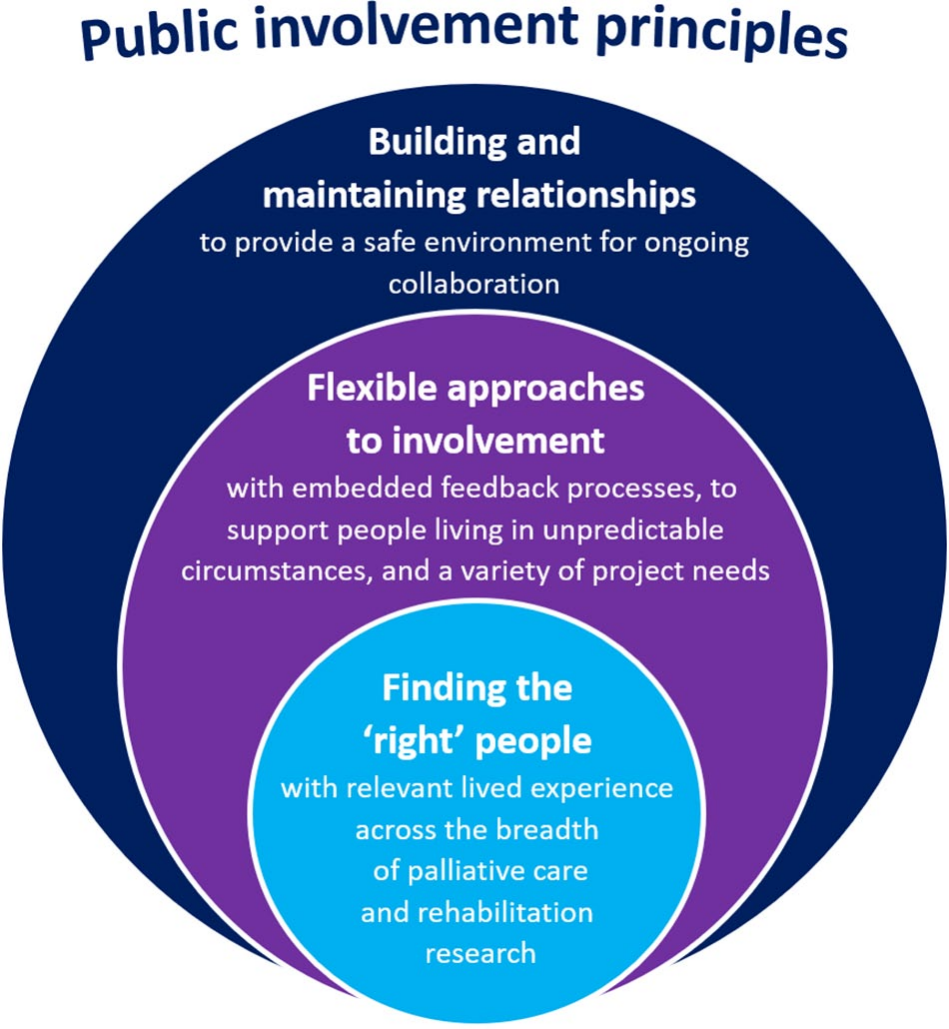
Revised principles to inform public involvement in palliative care and rehabilitation research.

Three themes were identified as crucial to successful public involvement in palliative care and rehabilitation research: (i) building and maintaining relationships; (ii) flexible approaches to involvement; and (iii) finding the ‘right’ people (see Figure 2).

These themes demonstrated what was working well within the current strategy and associated infrastructure, alongside areas for refinement and improvement, including several practical examples (Table 2).

**Table 2.** Quotes illustrating the themes in practice.

Theme	Illustrative quote
Building and maintaining relationships	*Within my own project, a lot of our PPI [patient and public involvement] members have been brought across, from other projects-to this project we’re working on now, and I think they get a sense of satisfaction and value that they are included in our steering group, they’ve been on lots of different panels, they’ve been part of our ethics panel, and they all keep coming back as well, because I think they, they have got an identity here and they have got a role, and I think they, everybody kind of acknowledges that and knows that and they feel very, happy to speak, so I get the sense that, within my own project, it-they, that there is a sense of value. (Researcher 13)*
Being flexible to enable involvement	*I think there has been a tendency to assume that you um, you know every aspect of research, and one of the best projects I’ve worked on is the one we’re just coming to the conclusion of, because I knew the terminology but I didn’t actually know having not done research about analysing data, about quantitative versus qualitative and it has been fantastic to actually get that training which has been a mixture of YouTube clips, and papers to read, you know b-but that’s good, because it has been a multi-media approach to learning those aspects of research, and that’s been really beneficial and to be actually immersed, it’s felt much more inclusive. (Public member 7)*
Finding the ‘right’ people	*We were discussing the results with one of the PPI [patient and public involvement] members who was a carer for a dementia patient. . .we were explaining how our next steps could be just analysing primary care and GP; then she said, ‘well, my mother never had a visit from the GPs, we almost never saw the GP’ - the person who was there all the time was District Nurses, and then we thought well, we should see what is going on with District Nurse, because it seems like, they are the ones who are taking care of the patients with dementia at the end of life, and that’s one of the things we will actually include now in our research. (Researcher 15)*

### Building and maintaining relationships

The public involvement strategy emphasised early involvement and promoting the contribution of public members, but these had become part of a wider theme of building and maintaining relationships. High-quality relationships were characterised by flexible methods of communication, early involvement, infrastructure to support relationships, continuity and emotional support.

Overall, public members felt relationships were managed well. Frequent and open communication channels and researchers extending opportunities to be involved made public members feel included, supported and valued, and created a sense of continuity.

*. . .you very much involve people, you let them know what opportunities there are, those are circulated and communicated, ah-and sometimes you reach out to individuals knowing their skill set, and say you know ‘would you be interested in this’ opportunity, and so I feel very valued (Public member 7)*

Offering multiple formats of involvement was key to initiating and sustaining relationships, enabling individuals to tailor involvement to their needs. Public members living with chronic illness and/or managing caring responsibilities valued the availability of virtual methods of communication and information transfer. This allowed them to be involved in key project meetings if unable to attend face-to-face and receive key documents without delay, minimising the burden of involvement and increasing accessibility.

*. . .without the skype and teleconferencing, this might have been a non-starter for me to be honest because I’ve got an illness. . .that’s got its limitations, I’ve got caring responsibilities . . .and, I am really busy that if I was having to come to London all the time, I think it would be too much. (Public member 6)*

However, some public members reiterated the importance of face-to-face meetings, which were perceived to enable higher quality communication, and the availability of hard copy documents to support readability.

*The authenticity of the information you get. . .face to face I think is really important. . . I think you learn more and you’re able to modify your views more if you want to when you hear other people sitting around a table. . . I think the quality of that information that the researchers pick up is probably of a higher quality than what you might get from emails and online. (Public member 4)*

Well my reading email is on my phone, so it’s a nightmare because, I’ve got a laptop but, I’m only half way getting it set up, cause I am not e-minded, so, to me, a piece of paper to read it is, how I like it. (Public member 2)

Early involvement in a research study remained key and was perceived by public members to foster more collaborative relationships. This included involvement activities pre-funding, such as developing research questions, plus early post-funding activities including reviewing study protocols and attending ethics committee meetings. At this stage, public members felt they could provide critical input and shape research in terms of its acceptability and relevance. Participants commented that this had improved over the period in which the strategy had been introduced.

*You’ve got your researchers coming in now, talking about their project pre-funding. . . so at very early stages. . . it’s a bit more like co-design, and there’s more personal involvement . . .so, it is collaborative from the beginning . . . and that is just unbelievably important, because it shapes where you go (Public member 4)*

There were also challenges of involving people early. Researchers found it difficult to manage the disappointment and expectations of public members if grant applications were unsuccessful. Early involvement was also limited by researchers’ uncertainty around how to fund public members’ time at this stage and limited awareness of funding available from local organisations to support such activities. Public members also felt that more transparent discussions about their role(s) and time/resource required for the project were needed during grant development. This would allow public members to consider their involvement in line with potential disease trajectory and/or caring responsibilities.

*It would be really good to get the, er, a magnitude er of, of the project, is it a six-month’s program, is it a six-year’s program, it just helps to understand how much involvement is there going to be, and, and, you know, am I going to be alive in that period, er, because that’s important. . . (Public member 3)*

Having suitable infrastructure in place to support researchers and public members, alongside increasing awareness of external funding and support, was perceived as key to overcoming some of these challenges. At a department level, the core institute public involvement group, public involvement workshops and the public involvement online forum were key to facilitating early involvement.

*. . . at a structural level. . . I can use the Dragon’s Den, or I can use the award-winning web-based platforms, or I can just ask a colleague and they have a database of people. . .-that sort of infrastructure, as a researcher, is amazing and I’m really grateful cos I think it really helps (Researcher 17)*

Challenges relating to maintaining relationships were also seen towards the end of projects. Some public members felt ‘abandoned’ when projects ended, and researchers struggled with meeting public members’ expectations about continuing project activities when funding had ceased, and often, employment contracts linked to the project were ending.

*. . . I’ve immersed myself in a project and then when it comes to end I can feel a little bit abandoned, and that’s something about drawing things to a close. . . especially in a project where you’ve really enjoyed it. . .and you, erm, sort of, got on well with the other PPI [patient and public involvement] reps and the researchers. You don’t really wanna see it disband. . . (Public member 6)*

. . . I feel like-r-we’re coming to the end of our project now, and, we have finite resources . . . funders saying ‘the money’s gonna stop at this point,’ and our PPI [patient and public involvement] are really, really great at ideas . . .and they really want to kind of think about the bigger picture which drives us forward . . . but I find myself caught in a tension between, what’s realistic, and what they want to do.. . .I can’t really commit to anything beyond this project. (Researcher 3)

Further integration of public members into the research institute, and core public involvement activities, rather than just project-specific involvement, was suggested to enable continuity when projects ended.

*My first experience here was just ah-straight in to um the meeting room. . .you were just straight into that project and it was very much that little silo that you worked in, and it was a long long time down the line, coming to other events, where I learnt about who was here, and get a whole feeling and sense of how the institute actually runs in an entity, rather than just coming in on that tiny little bit. (Public member 7)*

. . . putting them into the institute level, full of PPI [patient and public involvement] members. . .so their activities shouldn’t kind of end with a project, or their involvement shouldn’t end with a project, it should continue. I think that’s the way they’re gonna feel sort of valued. (Researcher 19)

Finally, public members suggested that further emotional support should be considered to maintain relationships, with the sensitive nature of palliative care research often meaning that involvement could require discussing emotional or distressing experiences. This could include offering a debrief before or after project meetings to ensure any concerns are communicated and discussed.

*. . .it can bring up a lot of memories from the past, and ah emotions, and that can be quite difficult to manage personally, because you might cope with it in that meeting or that setting, but then it sets off a whole train of thought and sort of sad reflections when you leave that meeting, and um, the impact can stay with you, especially reading transcripts, you need to be very mindful of that, and you know, protect yourself. (Public member 7)*

### Flexible approach to involvement

Consistent with the core principles of the public involvement strategy, a flexible approach to involvement remained important. Researchers used a variety of communication methods, and utilised infrastructure such as public involvement workshops and the online forum to support flexible involvement.

*We have adopted a variety of ways to involve them [Patient and Public Involvement members], for example skype, and telephone call. And even, for example, they haven’t, been able to make the meeting, we always send them the newsletters or, our meeting minutes, for them to ev-still like involve in a later subsequent discussion. (Researcher 9)*

However, discussions also highlighted how researchers needed to think in more flexible and meaningful ways about involvement for their individual projects. Researchers often took a standardised approach to involvement; whereby public members were being included at every stage of the research. However, doing so, without considering the aim or purpose of involvement at each stage was problematic.

*Potentially, we do a bit of a disservice sometimes when we just include them, without thinking about why, and I’ve definitely been in a room before, including on my own project, when there have been PPI [patient and public involvement] people in the room but I’m not really sure what the purpose is . . .it really hasn’t been beneficial. (Researcher 16)*

Researchers agreed that there was a need to tailor approaches to involvement to align with the study design and the skills, expertise and development needs of public members involved. For example, a primary data collection study may find it beneficial to have input from public members regarding the recruitment strategy and participant information materials, whereas routine data projects would not require this type of input and involvement may be focused more on, for instance, dissemination of project findings.

*. . . it makes sense to probably have a slightly different sort of role and format for PPI [patient and public involvement] within secondary analysis because, you already have the data and the data is there, a lot of the input is already, you’ve cut that off, and if you’re collecting primary data, it’s a lot more that, they, you know, that’s really when you need to engage them and so on and so forth. Erm, so it seems to me like, now we’re thinking of it and coming up with ideas and saying, actually, the system should be s-tailored to, I suppose, the study type. (Researcher 14)*

Having opportunities in meetings or workshops to share good practice and resources, including examples of public involvement across projects, was suggested by researchers as a useful way to build knowledge in different approaches to involvement, fostering greater flexibility.

*I know that there’s a wealth of ways that people have done that [patient and public involvement] here erm, but as someone who’s new, it’s like ‘ok, ah, exactly how am I gonna do that?’ . . .and what’s the right way to do it? . . . and it might be different for quant and qual. (Researcher 6)*

So sharing that, I guess sharing what’s worked, what we’ve done and . . . (Facilitator 1)

Yeah, what’s already known. And then it could be added to as we move forward and pick up new ways to do things. (Researcher 6)

Developing clear aims and plans for involvement in collaboration with public members at an early stage was also key for ensuring involvement was tailored and meaningful. Further, reflecting on and evaluating whether the initial aims of involvement were being met throughout the project, and adapting approaches flexibly was important.

*. . .have all the objective, including the PPI [patient and public involvement] objective been met? . . . when you’re writing the report, concluding . . .you need to address, ‘this was raised at the, at t-as-one of the aims or, an objective, and this is what was concluded,’ . . . (Public member 3)*

. . .so making a bit more explicit I guess almost the aim of your patient and public involvement and, whether that worked out . . . (Facilitator 1)

. . . absolutely, yes, and it, in some cases, you may have been able to address, and in some cases, you may not have been, but at least an acknowledgement of that, and, explaining the reason why you could not meet the objectives of PPI [patient and public involvement], ahh, will at least emphasise that, some more work needs to be done. . . (Public member 3)

Participants agreed that feedback (from researchers to public members, and vice versa) played an important role in this. However, public members highlighted that current provision of feedback across projects varied. This inconsistency may be explained by researchers’ uncertainty on how to capture the difference and impact of involvement. Participants noted the more intangible benefits of involvement, which is often challenging to record and quantify. One public member also highlighted how the impact of involvement on research, while sometimes immediate, often produces a domino effect.

*I would say that, sometimes the PPI [patient and public involvement] member just helps reframe a conversation in a slightly different way, and you, it’s a little bit intangible, the benefit (Researcher 17)*

. . .because that’s the thing with PPI [patient and public involvement], it sometimes doesn’t have an immediate effect on the project that you’re dealing with now, but because other projects follow on, sort of like a domino effect, you never know what you say at one meeting, is going to have an effect several projects down the line. . .(Public member 6)

Researchers suggested a need to standardise and embed feedback processes throughout the research cycle, including opportunities to reflect on the contribution of public members, whether the initial aims of involvement have been achieved, and if any improvements were required to facilitate involvement.

*it’s that process of feeding back . . .and I think, if I was to do that more regularly, that would be a good way of being more mindful about how they [public members] have changed, projects over time (Researcher 18)*

### Finding the ‘right’ people

Participants emphasised the importance of engaging the ‘right’ people for particular research projects, referring to those who had lived experience relevant to the study population, including experience of specific conditions or health services being investigated. While this was not considered within the current strategy, participants perceived it as key to ensuring meaningful involvement and enhancing the acceptability and relevance of research. Despite offering flexible methods of involvement, researchers were regularly not collaborating with public members who had experience sufficiently relevant to their project, often relying on the core public involvement group instead. This also led to the same public members often being involved across projects, limiting the inclusion of new perspectives within the research.

*I think it also comes down to actually making the effort to, to ensure you’ve got the ‘right’ people . . . I think it’s about not being complacent that, you put an advert out and, four people reply so it’s like. . . great you’ve got four people. . . Have you got the ‘right’ people? (Researcher 7)*

Participants suggested that more should be done to diversify and expand the pool of public members to ensure it reflected the diversity of people which palliative care serves. This included actively engaging with individuals with different conditions, different experiences and those currently not involved in research. Establishing links with wider groups and networks was perceived as one way to enhance diversity, alongside developing resources to support researchers when identifying people to involve.

*. . . it’s principally the, the people who are involved i-it’s trying to get all levels of society involved in PPI [patient and public involvement], and I know that’s difficult, and I know it can be unbelievably frustrating - I’ve watched it, but I think if you want to do, a k- a kind of – if you want to be inclusive. . . you need to do-be doing that. (Public member 4)*

So, diversifying and growing the group so it’s flexible in lots of different directions at different points, that reflect the needs of the institute would be useful sometimes, or at least having the resource to know where to go. . .. (Researcher 12)

## Discussion

Due to the nature of palliative care and rehabilitation research, public involvement requires particular consideration of how we build and maintain relationships, work flexibly, and find the ‘right’ people to work alongside. Building and maintaining relationships was essential for providing a safe environment to discuss the emotionally sensitive research topics often prevalent in palliative care and rehabilitation research. Flexible approaches are also needed when involving those living in the complex and unpredictable circumstances of chronic and life-threatening illness. Opportunities for institution-level involvement (e.g. the workshops) alongside project-specific collaborations and infrastructure for in-person and online opportunities, plus open and responsive communication, facilitates this. However, participants noted a need for more careful focus on emotional support and flexible approaches that stem from iterative feedback (from the researchers to the public members, and vice versa). Purposeful consideration of how involvement may be tailored to a particular project, and the need to seek out the ‘right’ people to work collaboratively with were considered important. The ‘right’ people were considered as individuals with relevant lived experience of the health condition or service being researched.

In line with Chambers et al.’ s recent review,^11^ we did not find the principles of public involvement in palliative care research to be entirely different from other research fields. In fact, our data often reflected many elements of the eight themes identified in Chambers et al.’s review,^11^ and those listed in the broader UK Standards for Public Involvement in Research.^20^ Rather, our findings identified areas of particular focus when considering the specific context of palliative care and rehabilitation research, and how core strategy and infrastructure can influence these. For example, our findings particularly highlight the need to establish support systems for public members involved in research which may be emotionally demanding. Opportunities for emotional support (e.g. de-brief meetings,^22^ peer support^23^) should be considered and discussed with public members at the early stages of involvement, as a core part of building and maintaining relationships.

Similar to other studies, our findings showed the importance of the broad principle of flexibility to facilitate involvement.^11,24–26^ In palliative care, providing flexible methods of involvement (e.g. in-person, online) was seen to be essential to successfully collaborate with individuals living in complex and unpredictable circumstances.^12^ Our findings, however, also highlighted the need for more flexible approaches within specific projects to help facilitate meaningful involvement. Ensuring embedded feedback processes throughout the project would improve flexibility, by ensuring that involvement could be tailored and adapted over time in response to feedback by public members to researchers (or vice versa), and in response to the needs of the specific project. Similar to previous research, the provision of feedback within projects in this evaluation was variable.^27^

Greater attention to diversity in involvement in research has been emphasised widely^20,28^ and was raised as an important consideration in this evaluation. In palliative care and rehabilitation research, particular care is needed to ensure that those involved reflect the wide diversity of demographics and conditions which palliative care serves. Within research projects, greater attention is needed to find the ‘right’ people; those who have experience relevant to the study population. When done well, this can help improve the relevance and impact of research, but when done poorly this can result in missed opportunities. As such, allocating increased time and resources to this early stage of involvement may help researchers to reach out to a wider diversity of public experience, and involve those who have the most to offer within each project.

More widely in palliative care research, participants acknowledged that while areas of good practice exist locally, there is scope for wider sharing of experiences and learning both nationally and internationally. This would not only help reduce duplication, but also provide inspiration of novel approaches to involvement across different project types and institutions. A more collaborative approach to public involvement in palliative care and rehabilitation research across organisations may help progress the quality and extent of involvement, internationally. This might include sharing examples of consumer-led work, which is currently limited in palliative care research and practice,^29^ and working together across research groups to share expertise and increase reach.^30^ Having identified how well-intentioned principles (e.g. a focus on early flexible involvement) can lead to unintended consequences (e.g. blanket approaches to involvement without careful consideration of its purpose), ongoing critical reflection will be required. Ultimately, this may improve the quality and relevance of palliative care research for those it is intended to benefit.

### Strengths and weaknesses

We purposefully took a co-productive approach to this study, working in a team of researchers and public contributors. Involvement from the conception of the project allowed for joint input on the study design, ethical considerations and data collection tools. Public contributors’ understanding and experience of the topic being explored, public involvement, aided their involvement in the analysis and interpretation stage, and ensured the work was not limited to a researcher perspective.^31^ We used a mixture of focus groups and interviews (including a telephone interview) to facilitate flexible involvement of public members who were unable to attend a certain time or venue. Moreover, we strengthened our analysis and interpretation by incorporating recent models of public involvement within and outside of palliative care.^20,21^

Due to convenience sampling, using existing meetings, it was not possible to determine the response rate to the study. Although no-one who attended the meetings declined to participate in the study, individuals who did not attend the meetings may have experiences that differ to those who did contribute. Further, as the evaluation was undertaken by researchers familiar to participants, participants may have censored some of their responses. We note, however, that we received both positive and negative responses during focus groups and interviews. Finally, our study took place at a single research institute in the UK where the strategy was originally developed and implemented. Although we anticipate the core principles of successful involvement are likely to be similar, further work is needed to understand which elements of the processes and infrastructure of our public involvement strategy might need adaptations to different contexts.

## Conclusion

Palliative care research often involves emotionally sensitive topics and diverse populations of people living in complex and unpredictable circumstances. Public involvement in palliative care research therefore needs to prioritise building and maintaining relationships, flexible approaches to involvement, and finding those with relevant lived experience for specific projects. Taking a strategic approach to public involvement within a research institute can facilitate this, but ongoing critical reflection on such initiatives is essential to build on and sustain successes and minimise unintended consequences. Future work is needed to understand the strategies and resources required to support this approach to involvement in different palliative care and rehabilitation research contexts and support wider national and international development of public involvement in palliative care and rehabilitation research.

## Supplemental Material

Supplementary_File_1_-_Focus_Group_topic_guides_1 – Supplemental material for Patient and public involvement in palliative care research: What works, and why? A qualitative evaluation Click here for additional data file. Supplemental material, Supplementary_File_1_-_Focus_Group_topic_guides_1 for Patient and public involvement in palliative care research: What works, and why? A qualitative evaluation by Halle Johnson, Margaret Ogden, Lisa Jane Brighton, Simon Noah Etkind, Adejoke O Oluyase, Emeka Chukwusa, Peihan Yu, Susanne de Wolf-Linder, Pam Smith, Sylvia Bailey, Jonathan Koffman and Catherine J Evans in Palliative Medicine

Supplementary_File_2-_Coding_framework_2 – Supplemental material for Patient and public involvement in palliative care research: What works, and why? A qualitative evaluation Click here for additional data file. Supplemental material, Supplementary_File_2-_Coding_framework_2 for Patient and public involvement in palliative care research: What works, and why? A qualitative evaluation by Halle Johnson, Margaret Ogden, Lisa Jane Brighton, Simon Noah Etkind, Adejoke O Oluyase, Emeka Chukwusa, Peihan Yu, Susanne de Wolf-Linder, Pam Smith, Sylvia Bailey, Jonathan Koffman and Catherine J Evans in Palliative Medicine

Supplementary_File_3_-GRIPP2_Short_Form_Resubmission – Supplemental material for Patient and public involvement in palliative care research: What works, and why? A qualitative evaluation Click here for additional data file. Supplemental material, Supplementary_File_3_-GRIPP2_Short_Form_Resubmission for Patient and public involvement in palliative care research: What works, and why? A qualitative evaluation by Halle Johnson, Margaret Ogden, Lisa Jane Brighton, Simon Noah Etkind, Adejoke O Oluyase, Emeka Chukwusa, Peihan Yu, Susanne de Wolf-Linder, Pam Smith, Sylvia Bailey, Jonathan Koffman and Catherine J Evans in Palliative Medicine
