# Supplementary material for: Patient and public involvement in palliative care research: What works, and why? A qualitative evaluation
Source: Palliat Med. 2020 Sep 11;35(1):151–60. doi: 10.1177/0269216320956819 (PMC7797607; doi:10.1177/0269216320956819)
Supplement: Supplementary_File_1_-_Focus_Group_topic_guides_1 – Supplemental material for Patient and public involvement in palliative care research: What works, and why? A qualitative evaluation [file Supplementary_File_1_-_Focus_Group_topic_guides_1.docx]

**Supplementary File 1 - Focus Group topic guides (Public involvement member and researchers)**

# Topic Guide for Public Involvement Members

## Introduction

**We would like to start this** focus group **by asking if you could each introduce yourself by giving your name, how long you have been involved in research, and how your involvement came about?**

TOPIC AREA A – PURPOSE OF PATIENT AND PUBLIC INVOLVEMENT

Can you tell us what you think is the purpose and role of public involvement in research?

In your opinion, how does public involvement bring added value? Do you think that other public involvement representatives and members of the research team share this view?

## TOPIC AREA B – EXPERIENCES OF PATIENT AND PUBLIC INVOLVEMENT

**We would like to explore further your experiences of patient and public involvement**

What do you think are the best ways to enable public involvement to work well and develop?

Prompt: What needs to be in place? What has worked well for you previously? Is public involvement well-coordinated in your view?

Do you think opportunities to be involved, are flexible, varied and are receptive to people’s needs?

What do you think are the downsides to public involvement?

Prompt: In terms of time? Appreciation? Acknowledgement? Anything others? (i.e. negative ones – not worthwhile, tokenistic, too many commitments, waste of money)

What do you think could be changed to make the experience better?

Prompt: Are there any new innovations or mechanisms to improve the process?

Are there areas of the research process which you don’t feel sufficiently involved in, or don’t feel involved at all?

Prompt: Are there any areas of research you would like to be more involved in?

Do you feel fully recognised for your contribution as a public involvement member?

Prompt: In what ways are you recognised? Acknowledgement? Payment? Any others?

## TOPIC AREA C – IMPACT OF PATIENT AND PUBLIC INVOLVEMENT

In your opinion, does patient and public involvement improve the **relevance** of research? Can you explain how?

Prompt: Can you provide an example? (More patient centred? Provides carer perspective? Incorporates equality and diversity? Any others?)

In your opinion does patient and public involvement improve the **quality** of research? Can you explain how?

Prompt: Can you provide an example? (i.e. patient’s often see what is missing, as researchers may be too close to the topic)

Can you give me any examples of how a study you have been involved in has been changed because of PPI?

How do you judge whether public involvement has made a difference to the study you are involved in?

Have you been in a situation where public involvement did not work as expected? Can you tell me more about this?

Has being involved in research as a public, patient or family representative had an impact on you? Can you tell me more about this?

Is there any other impact that you think patient and public involvement leads to?

## TOPIC AREA D – FUTURE DEVELOPMENT

What formal or informal training and/or information regarding research and/or palliative care would be beneficial to you as public involvement representatives?

In your opinion, how can we make patient and public involvement *better*?

Prompts: More feedback? Further promotion of online forum? Extending opportunities to be a co-applicant and user led research? Any others?

Is there anything else we have missed or should have talked about?

# Topic Guide for Researchers

Introduction

**We would like to start this** focus group **by asking if you could each introduce yourself by giving your name, role and how long you have incorporated public involvement in your research.**

- Prompt: Can you tell me a little bit about how this way of working has developed for you?

TOPIC AREA A – PURPOSE OF PATIENT AND PUBLIC INVOLVEMENT

In your opinion, what is the purpose & role of public involvement in research?

Does PPI bring added value and if so, can you explain how?

## TOPIC AREA B – EXPERIENCES OF PATIENT AND PUBLIC INVOLVEMENT

**We would like to explore further your experiences of patient and public involvement**

What do you think are the best ways to enable PPI to work well, for both researchers and public involvement representatives?

Prompt: What needs to be in place? What has worked well for you previously? Is public involvement well-coordinated in your view?

What challenges, if any, have you faced when engaging patient and public involvement in your projects?

Prompt: Have there been any times when you have been unable to incorporate public involvement?

What do you think could be changed to make the experience better?

Prompt: More time? More resources? Better co-ordination? Better access to public involvement representatives? Any others?

Which areas of the research process are PPI members more involved with, and why do you think this is?

Prompt: Dissemination? Presenting? Evaluation? Any others?

Prompt: Are there any areas you would like them to be more involved in? How could we achieve this?

Do you think PPI members feel recognised for their contribution?

Prompt: How do you promote their contribution? Feedback? Acknowledgement? Payment? Any others?

## TOPIC AREA C – IMPACT OF PATIENT AND PUBLIC INVOLVEMENT

In your opinion does public involvement improve the **relevance** of your research? Can you explain how?

Prompt: Can you provide an example?

In your opinion, does public involvement improve the **quality** of your research? Can you explain how?

Prompt: Can you provide an example?

Can you give me any examples of how you have changed your study because of public involvement?

How do you judge whether public involvement has made a difference to your study?

Prompt: can you give me an example?

Prompt: Do you evaluate the public involvement at the end of the project?

Have you been in a situation where public involvement did not work as expected? Can you tell me more about this?

Has engaging with public involvement representatives had an impact on you?

Prompt: Do you feel confident about engaging with patient and public representatives within your research? Are you more keen to incorporate it in to the next project?

Is there any other impact that you think public involvement leads to?

Prompt: Improving public involvement strategy? Improving communication with the public? Any others?

Do you feel that PPI is fully embedded within the Institute? If so, how?

TOPIC AREA D – FUTURE DEVELOPMENT

What training and/or information surrounding public involvement would be beneficial to you and your research?

In your opinion, how can we make patient and public involvement *better*?

Is there anything else we have missed or should have talked about?
