# Supplementary material for: Patient and public involvement in palliative care research: What works, and why? A qualitative evaluation
Source: Palliat Med. 2020 Sep 11;35(1):151–60. doi: 10.1177/0269216320956819 (PMC7797607; doi:10.1177/0269216320956819)
Supplement: Supplementary_File_2-_Coding_framework_2 – Supplemental material for Patient and public involvement in palliative care research: What works, and why? A qualitative evaluation [file Supplementary_File_2-_Coding_framework_2.docx]

***Supplementary file 2 - Coding Tree***

| Name |
| --- |
| Building and defining relationships |
| Continuity |
| Fulfilling expectations |
| Integration into the institute |
| Relationship quality and respect |
| Role negotiation |
| Emotional support |
| Flexible mode of communication (e.g. remote options) |
| Timely communication |
| Transparency on project limits |
| Early involvement |
| Funding |
| Thinking or planning |
| Time |
| Flexible approaches to public involvement |
| Breadth and depth of integration |
| Tailored approaches to project public involvement needed |
| Thinking and planning |
| Level of integration is project specific |
| Training and development of public involvement members to facilitate integration |
| Providing feedback |
| Reflection and or evaluation |
| Enhancing quality of research |
| Recognition and promoting public involvement contribution |
| Finding the 'right' people |
| Increasing diversity and access |
| Flexibility supporting inclusivity or diversity of public involvement |
| Enhancing relevance and acceptability of research |
