# Supplementary material for: Patient and public involvement in palliative care research: What works, and why? A qualitative evaluation
Source: Palliat Med. 2020 Sep 11;35(1):151–60. doi: 10.1177/0269216320956819 (PMC7797607; doi:10.1177/0269216320956819)
Supplement: Supplementary_File_3_-GRIPP2_Short_Form_Resubmission – Supplemental material for Patient and public involvement in palliative care research: What works, and why? A qualitative evaluation [file Supplementary_File_3_-GRIPP2_Short_Form_Resubmission.docx]

***Supplementary file 3 - GRIPP2-SF checklist for PPI in research***

| **Section and topic** | **Item** |
| --- | --- |
| **1: Aim**  Report the aim of PPI in the study | The aim of public involvement throughout this project was to ensure that our research conduct and processes were acceptable and appropriate to patients/carers who were participating; ensure interpretation of findings were grounded in patient/carer experiences; improve clarity and reach of dissemination. |
| **2: Methods**  Provide a clear description of the methods used for PPI in the study | We took a co-productive approach to involvement through out the study, involving public contributors as members of the research team who had shared input and decision making at all stages of the project. We used a mixture of face-to-face methods (e.g. inclusion in project meetings) and remote methods (via telephone and email). Involvement was flexible, with some members being more involved in some study components than others, depending on their interests and time available. |
| **3: Study results**  Outcomes—Report the results of PPI in the study, including both positive and negative outcomes | Three public contributors were involved in the conception and development of this project. Two contributed to the initial funding application to support this work, and the third member joined for the first project meeting, where the project and plans going forward were discussed. Specifically, public contributors were involved in the following stages:  *Development of Protocol and ethics application:*   - Co-development and review of protocol - Review of application to ethics   *Data Collection*   - Co-developed and reviewed topic guides for researchers and public member focus groups - Suggested additional public member participants by including the option for teleconference/skype interview/focus group   *Consultation synthesis and interpretation*   - All public contributors read through transcripts, familiarising themselves with the data - Public contributors were involved in a consultation to develop coding framework where initial areas of interest were identified and then grouped into broader themes. - Public contributor (MO) double-coded a subset of the transcripts, after receiving informal training from HJ. A two-week period was allocated to ensure ample time to conduct the coding. MO’s involvement in the analysis, led to a greater understanding of the data. MO particularly highlighted areas which she thought would have arisen from the focus groups, but were missing from the data, and identified additional relevant themes, particularly from a patient and public perspective. Further MO has previously demonstrated interest in qualitative data analysis, so this provided an opportunity for MO to develop skills and knowledge in this area.   *Data Interpretation*   - Multiple teleconferences were held between MO and researcher (HJ) to start to summarise and identify key findings from the data. While this was time consuming, it did lead to a number of important findings, and highlighted the areas which we should emphasise including continuity and relationship management, and the long-term nature of impact for public involvement. - All three public contributors also provided their interpretation of the data via either email or in face-to-face meetings   Dissemination:   - Co-authoring paper, including intellectual contribution to main messages of the paper - Public contributor (MO) co-developed (PowerPoint presentation) and presented initial findings and interpretation to a wider group of public members and researchers at an internal public involvement workshop in London. The presentation was well received. - We also plan to work with our public contributors to disseminate our experience of working together on this project in a short article and news item (e.g. for the INVOLVE newsletter). We are also looking for opportunities to co-present findings from the evaluation with a wider audience, such as national conferences. - Public contributor (MO) has made critical decisions regarding responses to reviewer and editorial comments and has contributed to the revision of this manuscript. |
| **4: Discussion and conclusions**  Outcomes—Comment on the extent to which PPI influenced the study overall. Describe positive and negative effects | There have been multiple positive outcomes from public involvement in our study, in line with our aims:  *Ensure acceptable and appropriate research processes where patient/carers would be participating:* Involvement of our public contributors in reviewing the protocol and topic guide for both researcher and public member focus groups allowed us to be more confident that the questions we were asking were appropriate and acceptable. Public contributors were particularly helpful at providing prompts to questions, which were highly useful during the conduct of focus groups. Public contributors also suggested including the option for tele/videoconference participation in focus groups, which were added to the ethics application and protocol.  *Ensure interpretation of findings were grounded in patient/carer experiences:* Involving a public member to conduct the thematic analysis following one-to-one training ensured both a patient and public and researcher perspective guided interpretation. It was at this stage new insights were raised (e.g. how the long-term impact of public involvement is understood and appreciated by public members). Having all three public contributors included in data interpretation, also ensured that each had an opportunity to relate emerging findings to their real-life experiences (e.g. highlighting issues of continuity, emotional support offered by individual researchers and communication which they have identified from their own involvement)  *Improve clarity and reach of dissemination:* By being involved in the project and understanding findings and key messages, public contributors who are also involved with other organisations have started to share findings via word of mouth. This has led to further reach of our work and sharing of the value of conducting such an evaluation co-productively. Having public co-authors have also increased clarity of the paper, researcher terms such as ‘instances of silence’ have been clarified in text, for a more public audience. Public contributor (MO) has also reviewed the reviewer and editorial peer review comments and been involved in the revision of this paper. Public members have also suggested additional ideas for dissemination, including possible conferences and groups to target with dissemination. |
| **5: Reflections/critical perspective**  Comment critically on the study, reflecting on the things that went well and those that did not, so others can learn from this experience | We feel that this project, particularly the data analysis and interpretation component, benefited greatly from public involvement. As this was a multi-perspective evaluation it was important to have a balance of public and researcher views at this stage to ensure interpretation was not biased and consensus was reached from both perspectives. We found it was beneficial to have multiple public contributors engaged at the start of the project, as this gave flexibility for more or less involvement from individuals at different stages, in line with their interests, other commitments, and/or health. In line with this, the two public contributors who were involved heavily at the start, provided less input within the middle of the project.  While co-production does not have a set definition, our approach to co-production throughout the project encompassed forming a project team inclusive of both public contributors and researchers, with equal power and joint decision making and input. Taking this collaborative approach, aided the success of this project, with collaboration and a building of trust between public contributors and researchers from the conception of the project making it easier to aid involvement in later stages of the project including data analysis and interpretation.  Our public contributors have commented that they felt valued as part of the project team, leading to more rewarding and satisfying involvement, as well as ensuring personal development. This was particularly the case for MO, who was enthusiastic about developing skills in qualitative data analysis. MO has also reflected since the project, that these skills have been applicable to other areas of involvement. As the project was extremely relevant to those involved, public contributors often were contributing without prompt, and were keen to be involved in-depth through-out the project.  There has been ongoing conversation via an email thread during the life of this project which has ensured continuity and maintained momentum, which has ensured all felt involved at every step of the way. |
